# Enhancement of photosynthetic isobutanol production in engineered cells of *Synechocystis* PCC 6803

**DOI:** 10.1186/s13068-018-1268-8

**Published:** 2018-09-27

**Authors:** Rui Miao, Hao Xie, Peter Lindblad

**Affiliations:** Microbial Chemistry, Department of Chemistry-Ångström Laboratory, Uppsala University, Box 523, 751 20 Uppsala, Sweden

**Keywords:** *Synechocystis*, Cumulative titer, Cultivation condition, Metabolic bottleneck, Co-expression

## Abstract

**Background:**

Cyanobacteria, oxygenic photoautotrophic prokaryotes, can be engineered to produce various valuable chemicals from solar energy and CO_2_ in direct processes. The concept of photosynthetic production of isobutanol, a promising chemical and drop-in biofuel, has so far been demonstrated for *Synechocystis* PCC 6803 and *Synechococcus elongatus* PCC 7942. In *Synechocystis* PCC 6803, a heterologous expression of α-ketoisovalerate decarboxylase (Kivd) from *Lactococcus lactis* resulted in an isobutanol and 3-methyl-1-butanol producing strain. Kivd was identified as a bottleneck in the metabolic pathway and its activity was further improved by reducing the size of its substrate-binding pocket with a single replacement of serine-286 to threonine (Kivd^S286T^). However, isobutanol production still remained low.

**Results:**

In the present study, we report on how cultivation conditions significantly affect the isobutanol production in *Synechocystis* PCC 6803. A HCl-titrated culture grown under medium light (50 μmol photons m^−2^ s^−1^) showed the highest isobutanol production with an in-flask titer of 194 mg l^−1^ after 10 days and 435 mg l^−1^ at day 40. This corresponds to a cumulative isobutanol production of 911 mg l^−1^, with a maximal production rate of 43.6 mg l^−1^ day^−1^ observed between days 4 and 6. Additional metabolic bottlenecks in the isobutanol biosynthesis pathway were further addressed. The expression level of Kivd^S286T^ was significantly affected when co-expressed with another gene downstream in a single operon and in a convergent oriented operon. Moreover, the expression of the ADH encoded by codon-optimized *slr1192* and co-expression of IlvC and IlvD were identified as potential approaches to further enhance isobutanol production in *Synechocystis* PCC 6803.

**Conclusion:**

The present study demonstrates the importance of a suitable cultivation condition to enhance isobutanol production in *Synechocystis* PCC 6803. Chemostat should be used to further increase both the total titer as well as the rate of production. Furthermore, identified bottleneck, Kivd, should be expressed at the highest level to further enhance isobutanol production.

**Electronic supplementary material:**

The online version of this article (10.1186/s13068-018-1268-8) contains supplementary material, which is available to authorized users.

## Background

Isobutanol is a branched-chain, four-carbon alcohol used in various manufacturing industries. It is recognized as a promising gasoline additive candidate due to its higher energy content, lower vapor pressure, and lower water solubility compared to ethanol, the most commonly used gasoline supplement in the market today [1]. Isobutanol can be used as a drop-in fuel in the petroleum infrastructure without causing engine and pipeline corrosion [2]. All these advantages make isobutanol an attractive chemical. In addition, isobutanol can be produced via biological routes.

The 2-keto acid pathway is a branched-chain amino acids synthesis pathway, and the 2-ketoisovalerate produced via this pathway can be decarboxylated by an α-ketoisovalerate decarboxylase into isobutyraldehyde and then further converted to isobutanol by an alcohol dehydrogenase. This isobutanol biosynthesis pathway has been widely engineered into various microorganisms, such as *Escherichia coli* (*E. coli*) [3–8], *Saccharomyces cerevisiae* (*S. cerevisiae*) [9–11], *Bacillus subtilis* (*B. subtilis*) [12], *Corynebacterium glutamicum* (*C. glutamicum*) [13, 14], and cyanobacteria [15–18]. Specifically, in the unicellular cyanobacterium *Synechocystis* PCC 6803 (referred hereafter as *Synechocystis*), it was demonstrated that a heterologous expression of an α-ketoisovalerate decarboxylase (Kivd) from *Lactococcus lactis* (*L. lactis*) results in an isobutanol and 3-methyl-1-butanol (3M1B)-producing strain [17]. In addition, the heterologously expressed Kivd was identified as a bottleneck in this isobutanol synthetic pathway, e.g., isobutanol production level correlated positively with Kivd expression levels. Moreover, an endogenous alcohol dehydrogenase in *Synechocystis* showed a significant high activity towards isobutanol production [17]. In a following study, the substrate-binding pocket of Kivd was engineered to gain higher activity towards isobutanol rather than 3M1B production. ST (Kivd^S286T^), with a replacement of serine-286 to threonine, showed both the highest increase in total catalytic activity and a preferential shift towards isobutanol production [18].

However, the isobutanol titer observed in all the engineered *Synechocystis* strains still remained low and it was unclear whether other metabolic bottlenecks (besides Kivd), or the culture condition employed, were limiting the isobutanol synthesis. Herein, in the present study, we investigate how to adjust the cultivation conditions to further enhance isobutanol production in *Synechocystis*. In addition, we address the existence of the other potential metabolic bottlenecks than Kivd in the isobutanol synthesis pathway when introduced into the *Synechocystis* cells.

## Methods

### Strains and cultivation conditions

*Escherichia coli* strain DH5αZ1 (Invitrogen) was used for cloning and conjugation work. The cells were grown at 37 °C in LB medium (agar or liquid) supplemented with 50 μg ml^−1^ kanamycin (Sigma-Aldrich). The glucose-tolerant *Synechocystis* strain was used in this study. *Synechocystis* seed cultures were grown under 35 μmol photons m^−2^ s^−1^ at 30 °C in BG11 with addition of 50 μg ml^−1^ kanamycin. Experimental cultures were inoculated as OD_750_ = 0.1 with a total volume of 25 ml in BioLite 25 cm^2^ plug-sealed tissue culture flasks (Thermo Fisher Scientific). The medium used was BG11 medium with addition of 50 μg ml^−1^ kanamycin and 50 mM NaHCO_3_ (Sigma-Aldrich). For examining different light intensities, the flasks were shaken horizontally at 120 rpm, at 30 °C, under 15, 50, and 100 μmol photons m^−2^ s^−1^, respectively. Light was measured using LI-190SB Quantum sensor. For the examining of different pH adjustment methods, 50 μmol photon m^−2^ s^−1^ light-intensity growth condition was employed. For HEPES-buffered cultures, 50 mM HEPES (Sigma-Aldrich) was added into the flasks every second day with the supplement media. For the HCl-titrated cultures, 37% HCl (Sigma-Aldrich), maximal 40 μl, was added every day to adjust the pH of the culture between 7 and 8. pH was measured using MColorpHast™ pH-indicator strips (pH 6.5–10) (Merck). 2 ml of culture was sampled from each flask every second day for OD_750_ and products measurements, and replaces with 2 ml fresh BG11 medium with addition of 50 μg ml^−1^ kanamycin and 500 mM NaHCO_3_. For the tolerance test, mid-log-phase pEEK2-ST cells were washed with fresh BG11 media and resuspended in BG11 media with 50 mM NaHCO_3_ to OD_750_ = 0.5 in plug-sealed tissue culture flasks (Thermo Fisher Scientific). Three isobutanol concentrations (321 mg l^−1^, 642 mg l^−1^, and 962 mg l^−1^) and two isobutyraldehyde concentrations (632 mg l^−1^ and 948 mg l^−1^) were examined. OD_750_ of the cultures were measured after 24 h.

### Plasmid construction

The self-replicating plasmid pEEK2-ST from our previous study [18], containing a strong constitutive promoter P*trc*_core_ and the engineered Kivd named ST, was used as template to make further constructs in this study.

### Transformation of *Synechocystis* via conjugation

*Escherichia coli* cargo cells and *E. coli* HB101 helper cells with the plasmid pRL443-AmpR were grown at 37 °C. The overnight cultures were centrifuged at 3000 rpm for 5 min and resuspended in fresh liquid LB medium without antibiotics. Wild-type *Synechocystis* cells (200 μl) were mixed with 1 ml cargo cells and 1 ml helper cells, and the mixture was incubated under 100 μmol photons m^−2^ s^−1^ at 30 °C for 1.5 h before being spread on a filter on BG11 agar plate for another 48 h incubation.

For colony selection and maintenance, filters were changed onto new BG11 agar plates with 50 μg ml^−1^ kanamycin. Gene-specific primers were used to analyze the colonies and the correct colonies were inoculated into 6-well plates for further use.

### Crude protein extraction and SDS-PAGE/western immunoblot

Proteins were extracted from day 2 cultures; cells were harvested by centrifugation at 5000 rpm, 4 °C, for 10 min. The pellet was washed in 2 ml PBS and recollected by centrifugation, 5000 rpm, 4 °C, for 10 min, and resuspended in 200 µl PBS with Protease Arrest (GBioscience). This mixture went through freeze–thaw process and was disrupted by acid-washed 425–600 μm-diameter glass beads (Sigma-Aldrich) using a Precellys-24 Beadbeater (Bertin Instruments), program 3 × 30 s. Centrifugation was performed twice at 12,000 rpm, 4 °C, 1 min each, to get a clean supernatant-containing soluble proteins. Protein concentration was determined by the DC protein assay (Bio-Rad). 5 μg soluble proteins from each strain were separated by SDS-PAGE, using Mini-PROTEAN TGX TM gels (Bio-Rad), and transferred to PVDF membrane (Bio-Rad). Anti-Strep-tag II (abcam) antibody was used to detect Strep-tagged Kivd through the standard techniques, while anti-ATPase western immunoblot was done on the same samples as loading control. This western immunoblot was repeated three times for determining the relative Kivd expression level in all strains.

### Optical density measurement and product quantification

Optical density was measured every day in 96-well plates using a micro-plate reader (HIDEX, Plate Chameleon). Every second day, 2 ml culture was sampled from each flask and centrifuged at 5000 rpm, for 10 min. 1305 μl of the supernatant was transferred into a 15 ml screw cap tube, and mixed with 45 μl 3000 mg l^−1^ internal standard (1-pentanol) and 450 μl extraction solvent dichloromethane (DCM). The mixture was shaken on Multi-Tube Vortexer VX-2500 (VWR) at maximum speed for 5 min and then centrifuged at 5000 rpm, 4 °C, for 10 min. The DCM phase (bottom) was transferred into 1.5 ml clear glass gas chromatography (GC) vials (VWR). The extracted samples were analyzed on a PerkinElmer GC 580 system equipped with a flame ionization detector and an Elite-WAX Polyethylene Glycol Series Capillary column, 30 m × 0.25 mm × 0.25 μm (PerkinElmer). Nitrogen was the carrier gas, with 10 ml min^−1^ flow rate. The temperatures of injector and detector were 220 °C and 240 °C, respectively. The initial oven temperature was 50 °C and then raised to 100 °C with a rate of 10 °C min^−1^ followed by a rise to 180 °C with a rate of 20 °C min^−1^. The GC results were analyzed using TotalChrom Navigator version 6.3.2.

## Results and discussion

### Modification on the cultivation condition

**Fig. 1:**
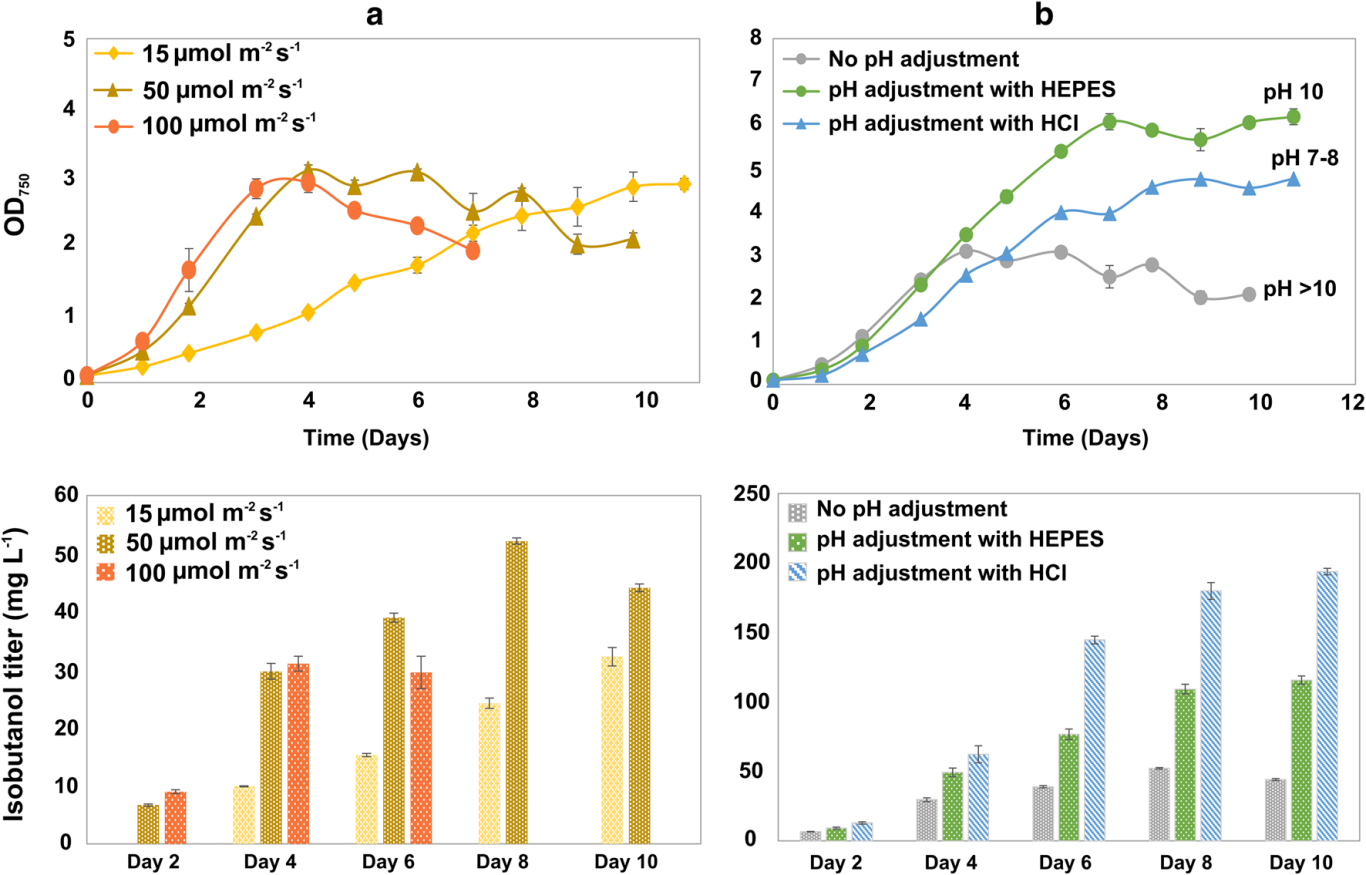
Growth and isobutanol titer produced from engineered *Synechocystis* PCC 6803 strain pEEK2-ST in different cultivation conditions. **a** Growth and isobutanol production titer of the cells grew under 15 μmol photons m^−2^ s^−1^, 50 μmol photons m^−2^ s^−1^, and 100 μmol photons m^−2^ s^−1^ light intensities without any pH adjustment. **b** Growth and isobutanol production titer of the cells grew under 50 μmol photons m^−2^ s^−1^ with different pH adjustments. The target pH range for the pH adjustment, using either HEPES or HCl, was between pH 7 and 8. The pH labeled on the growth curve was the final pH measured in the end of the cultivation. Results represent the mean of three biological replicates; error bars represent standard deviation

In the present study, we attempted to investigate how to improve cultivation conditions for enhanced isobutanol production in *Synechocystis*. A strain, pEEK2-ST, solely expressing Kivd^S286T^ on a self-replicating vector pEEK2, generated for enhanced isobutanol production in our previous study [18] was used for the cultivation experiments. Initially, we examined growth and isobutanol production under different light intensities (Fig. 1a). All experimental cultures were grown in plug-sealed tissue culture flasks to avoid product evaporation. We inoculated the cultures as OD_750_ = 0.1 from seed culture grown in cotton-cap E-flasks under 35 μmol photons m^−2^ s^−1^.

In the first 3 days, cells under 100 μmol photons m^−2^ s^−1^ grew significantly faster than under the other two light intensities examined. However, from day 4, cells under 100 μmol photons m^−2^ s^−1^ became yellowish and the OD_750_ started to drop quickly with a completely bleached phenotype at day 7. Moreover, the isobutanol titer observed in cultures under 100 μmol photons m^−2^ s^−1^ did not show any significant superiority compared to the cultures under lower light intensities. In addition, the empty vector control strain showed similar growth trends under these three different light conditions (Additional file 1: Figure S1). Moreover, in an isobutanol and isobutyraldehyde tolerance test, we did not observe a significant effect on cells growth in media with maximal 962 mg l^−1^ isobutanol (Additional file 1: Figure S2). This indicates that the phenotype of the cells under different light intensities does not correlate with the isobutanol production levels. The unhealthy phenotype of the cells under 100 μmol photons m^−2^ s^−1^ after day 4 was most likely due to the potential photoinhibition from the relative high light [19–22].

We observed the highest OD_750_ and isobutanol production from cells grown under 50 μmol photons m^−2^ s^−1^. These cells also maintained a longer steady state than the cells under 100 μmol photons m^−2^ s^−1^ and the isobutanol in-flask titer reached 52 mg l^−1^ at day 8. Furthermore, the cells under 15 μmol photons m^−2^ s^−1^ showed the slowest growth rate with an isobutanol in-flask titer of 32 mg l^−1^ at day 10 when the cultures went into steady stage. Based on these observations, we selected 50 μmol photons m^−2^ s^−1^ as light intensity for further investigations.

Since we grew the cells in sealed flasks, the supplementary of NaHCO_3_ every second day became the carbon supply for photosynthesis. In addition, *Synechocystis* cells in the early stationary phase naturally alkalize their living environment to pH around 10. As a consequence, the alkaline condition generated by both NaHCO_3_ and cells may cause changes in carbon equilibrium in the growth media or cell metabolism. Therefore, we compared two widely used pH adjustment approaches, HEPES buffer and HCl titration, where, in the control group, cells were grown without any pH adjustment.

The control group showed the least growth, fastest cell death, and the lowest isobutanol production compared to the cultures with pH adjustment. HEPES-buffered cultures showed the highest OD_750_, while the HCl-titrated cultures showed the highest isobutanol production (Fig. 1b). The dramatic differences between the cultures with and without pH adjustment may indicate that, in the sealed cultivation system applied, pH plays an important role to affect carbon supply. When the pH was above 10, most of the carbon existed in the form of CO_3_^2−^ in the media, which cannot be up taken by the cells. When the pH in the culture was adjusted, the carbon equilibrium shifted forward to generate relatively more HCO_3_^−^ and CO_2_. At this stage, the cells can assimilate more carbon, resulting in enhanced growth and isobutanol production. However, the HEPES-buffered cultures grew to a higher OD_750_ than the HCl-titrated culture, though the pH in the later cultures was closer to the theoretical optimal pH for *Synechocystis* [23] (Fig. 1b). This may be due to HEPES being much milder than HCl and better for maintaining enzyme structure and cell physiological characters [24, 25].

**Fig. 2:**
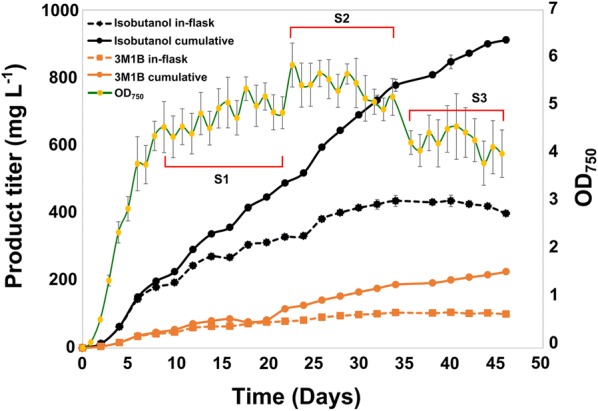
Growth, isobutanol/3M1B in-flask and cumulative titer observed in a long-term cultivation experiment. The OD_750_ was measured every day and product titer was measured every second day. S1, S2, and S3 represent different periods in the steady-state phase. Results represent the mean of three biological replicates and error bars represent standard deviation

To our surprise, the HCl-titrated cultures stayed in stationary phase(s) for long time, and we continuously observed isobutanol production from these cultures until day 46. The cultivation was terminated when the isobutanol in-flask titer had dropped for three continues measurements. A maximal in-flask isobutanol titer of 435 mg l^−1^ was observed at day 40 when the OD_750_ was 4.7 (Fig. 2). This titer is 1.5 times higher than the reported net isobutanol concentration observed in another isobutanol producing *Synechocystis* strain under mixotrophic cultivation condition at an OD_730_ above 12 [26]. After day 40, the isobutanol in-flask titer started to drop, the isobutanol produced between two measurements was not enough to overcome the dilution caused by the sampling of 2 ml culture and the supplement of 2 ml fresh media every second day. In the end of 46 days culturing, the in-flask isobutanol titer was 397 mg l^−1^ and the cumulative titer was as high as 911 mg l^−1^. Moreover, we also calculated the in-flask and cumulative titer of the by-product 3-methyl-1-butanol (3M1B). A maximal in-flask titer of 105 mg l^−1^ was observed at day 40 with a final cumulative titer of 225 mg l^−1^ at day 46. In addition, we divided the 46 days cultivation period into growth and steady-state phases, the isobutanol production rate in the growth phase was significantly higher than that in any of the steady-state phases (Table 1). The highest isobutanol production rate of 43.6 mg l^−1^ day^−1^ was observed between days 4 and 6, coinciding with maximal growth (Table 1 and Fig. 2). This demonstrates that maintaining the culture in the growth phase may be an optimal approach to further enhance isobutanol production. Therefore, a chemostat cultivation should be used in the future.

**Table 1:** Isobutanol production rates in the different phases of the long-term cultivation of engineered *Synechocystis* PCC 6803

	Time	In-flask (mg l^−1^ day^−1^)	Cumulative (mg l^−1^ day^−1^)
Growth phase	Days 2–10	22.6 ± 0.4	26.6 ± 0.3
Steady-state phase (total)	Days 10–46	5.7 ± 0.2	19.0 ± 0.3
Steady-state phase (S1)	Days 10–22	11.2 ± 0.5	21.9 ± 0.4
Steady-state phase (S2)	Days 23–35	10.3 ± 1.5	25.9 ± 1.8
Steady-state phase (S3)	Days 36–46	− 4.2 ± 0.7	12.9 ± 0.8
Maximal rate	Days 4–6	41.1 ± 3.5	43.6 ± 3.3

### Further investigation on the isobutanol synthesis pathway

**Fig. 3:**
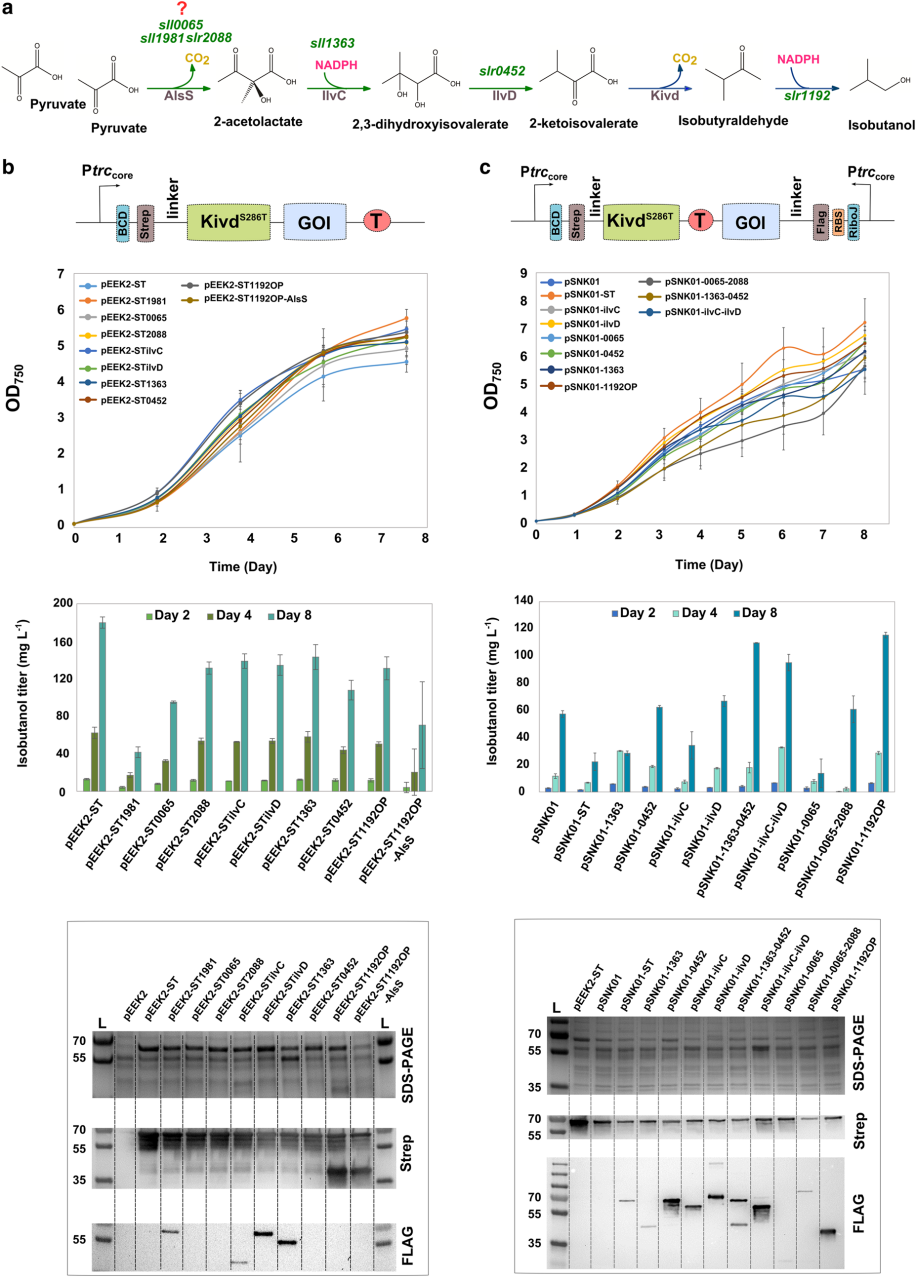
Schematic overview of the isobutanol synthesis pathway, the genetic constructs for identifying respective bottleneck, and the results from the engineered *Synechocystis PCC 6803* strains containing different constructs. **a** Isobutanol synthesis pathway. The heterogenous enzymes are colored in brown: acetolactate synthase (AlsS) from *Bacillus subtilis* (*B. subtilis*), acetohydroxy acid isomeroreductase (IlvC), dihydroxy acid dehydratase (IlvD) from *E. coli*, and α-ketoisovalerate decarboxylase (Kivd) from *L. lactis*. The endogenous genes are colored in green. The gene-encoding alcohol dehydrogenase used here is the codon-optimized *slr1192* (*slr1192*OP). **b** Genetic concepts, growth curve, isobutanol titer, SDS-PAGE, and western immunoblot from the engineered strains containing single operon. *GOI* Gene of interest. Kivd^S286T^ and *slr1192*OP-encoded alcohol dehydrogenase were strep-tagged and the other genes were flag-tagged. BCD is the bicistronic design for providing more reliable expression. **c** Growth curve, isobutanol titer, SDS-PAGE, and Western blot from the engineered strains containing convergent-oriented operons. Kivd^S286T^ was strep-tagged and the other genes were Flag-tagged. RiboJ is a self-cleaving ribozyme for providing more reliable expression. Size of the proteins are: AlsS: 62 kDa, IlvC: 54 kDa, IlvD: 65 kDa, KivdS^286T^: 61 kDa, Sll1981: 60 kDa, Sll1363: 40 kDa, Slr0452: 59 kDa, Slr1192: 36 kDa, Sll0065: 21 kDa, Slr2088: 68 kDa

Despite modifying the cultivation condition, we also aimed to further investigate the isobutanol synthesis pathway to identify other potential metabolic bottleneck enzyme(s) than Kivd [18]. In *Synechococcus*, an additional heterologous expression of acetolactate synthase (AlsS) from *Bacillus subtilis* (*B. subtilis*), acetohydroxy acid isomeroreductase (IlvC) and dihydroxy acid dehydratase (IlvD) from *E. coli* (Fig. 3a) enhanced isobutanol production 2.5 times [16]. Therefore, in the present study, we examined the heterogenous AlsS, IlvC, and IlvD discussed above, and the corresponding endogenous enzymes in *Synechocystis*. Moreover, we also attempted to investigate the effect on isobutanol production when expressing an additional copy of the endogenous alcohol dehydrogenase (ADH) encoded by codon-optimized *slr1192*, since the native ADHs may not be sufficient to convert the enhanced level of isobutyraldehyde produced by Kivd^S286T^.

It was unclear whether *Synechocystis* has a native AlsS or only an acetohydroxy acid synthase (AHAS), which is a bi-functional enzyme catalyzing the reaction from pyruvate to acetolactate and also the reaction from pyruvate and α-ketoglutarate to α-acetohydroxybutyrate [27]. Thus, we attempted to overexpress the endogenous gene-encoding potential AlsS and AHAS in *Synechocystis* (Fig. 3a) to examine their effects on isobutanol production. We initially generated eight engineered *Synechocystis* strains (Additional file 1: Figure S3A) where each strain contained an operon-expressing Kivd^S286T^and one of the genes to be examined. Unfortunately, we could not get transformants for the strain containing Kivd^S286T^ and AlsS (*B. subtilis*). Similar result has been reported in *Clostridium cellulolyticum* (*C. cellulolyticum*); no transformants could be obtained when expressing this AlsS with high transcription level. This may be due to the high activity of AlsS [28]. Therefore, we constructed another strain-containing AlsS in the end of the operon-expressing Kivd^S286T^ and ADH (Additional file 1: Figure S3B).

The growth and isobutanol titer from the obtained engineered strains were compared to the control strain pEEK2-ST. The variations in isobutanol titer from the three biological replicates of strain pEEK2-ST1192OP-AslS were very high and no AlsS expression could be detected using western immunoblots from any of the replicates (data not shown). Besides, isobutanol production from strain pEEK2-ST1981 was five times lower than from the control strain pEEK2-ST (Fig. 3b). This could be interpreted as an evidence for the α-KGD function but not an AlsS function of the enzyme encoded by *sll1981,* since the overexpression of *sll1981* might direct more carbon flux towards the TCA cycle instead of isobutanol synthesis [29–32]. Surprisingly, strain pEEK2-ST still showed the highest isobutanol in-flask titer, which corresponded to its highest expression level of Kivd^S286T^ among all the examined strains (Fig. 3b). In addition, the expression of *sll0065, slr2088,* and *slr0452* could not be detected using anti-Flag tag western immunoblot. Thus, two hypotheses could be made: (i) there is no other bottleneck(s) than Kivd in the isobutanol synthesis pathway. Thus, the strain with the highest Kivd^S286T^ expression level showed the highest isobutanol production. (ii) There is other bottleneck(s), but could not be evaluated due to the low protein expression levels.

Therefore, to increase protein expression levels, we expressed Kivd^S286T^ solely in a P*trc*_*core*_BCD driven operon and the other gene(s) to be examined in a convergent-oriented operon driven by P*trc*_*core*_RiboJ (Additional file 1: Figure S3C). A double BioBrick terminator B0015 was applied between these two operons to avoid read through. The new control strain pSNK01 contains the operon-expressing Kivd^S286T^ and a convergent operon-expressing *ccdB*. The addition of the convergent-oriented operon successfully increased the expression levels of all the enzymes to be examined. Strains pSNK01-1363-0452 and pSNK01-ilvc-ilvD showed significantly higher isobutanol production than the control strain. However, strains pSNK01-1363 and pSNK01-ilvC showed only half of isobutanol production compared to the control, and strains pSNK01-0452 and pSNK01-ilvD did not show any improvement in isobutanol production (Fig. 3c). These results indicate that acetohydroxy acid isomeroreductase (IlvC) may be a bottleneck enzyme in the isobutanol synthesis pathway, but the intermediate metabolite that produced may have negative effects on the metabolism in the *Synechocystis* cells. Therefore, dihydroxy acid dehydratase needs to be co-expressed with acetohydroxy acid isomeroreductase for enhanced metabolic flux towards isobutanol production. Furthermore, compared to the control strain, strain pSNK01-1192OP showed more than five times higher isobutanol titer, which means that the additional copy of ADH increased the conversion of isobutyraldehyde to isobutanol. To increase the level of Kivd^S286T^, the strain pSNK01-ST containing two copies of Kivd^S286T^ was made. Unfortunately, this strain showed less than half of the isobutanol titer compared to the control strain (Fig. 3c). This may be due to the lower Kivd^S286T^ expression level in both operons compared to that in the control strain or some mutations might occur when the construct was conjugated into *Synechocystis* and the activity of Kivd^S286T^ was interrupted.

Strain pSNK01-0065 showed significantly lower isobutanol titer compared to all the other engineered strains, this may indicate that the protein encoded by *sll0065* is similar to the small subunit of the AHAS from *S. cerevisiae* which functions as the regulatory subunit that inhibits the catalytic subunit of AHAS in response to a feedback signal from excess levels of valine [33]. Interestingly, strain pSNK01-0065-2088 showed similar isobutanol titer but much lower OD_750_ compared to the control strain, meaning that the isobutanol production per cell was higher in strain pSNK01-0065-2088. This may confirm the function of *slr2088* as the catalytic subunit of AHAS in *Synechocystis*. Unfortunately, we were unable to obtain any transformants for strain pSNK01-2088, which may be due to the similar problem occurred when expressing AlsS from *B. subtilis*.

Notably, all the strains containing two convergent operons showed much lower Kivd^S286T^ expression levels and thereby much less isobutanol production than strain pEEK2-ST (Fig. 3c). This may be due to the transcriptional interference between two operons caused by the leakage of terminator, though the convergent-oriented operons were claimed as the best composition to have high expression level for the genes in both operons [34, 35]. Therefore, the results indicate that keeping a high expression level of Kivd^S286T^ is an essential requirement for high isobutanol production in engineered strains of *Synechocystis*. Expressing Kivd^S286T^ alone on a high-copy number self-replicating vector is so far the best option to obtain the highest expression in *Synechocystis,* whereas all the other genetic modifications may be done in the genome. Furthermore, genome-scale metabolic models should be applied in the future to further identify potential metabolic bottlenecks and enhance the carbon flux towards isobutanol production.

## Conclusions

In summary, the present study demonstrates the importance of applying suitable cultivation condition for enhancing isobutanol production in *Synechocystis*. The isobutanol titer was dramatically increased in cells cultivated under 50 μmol photons m^−2^ s^−1^ and regularly adjusted to a pH between 7 and 8. A cumulative isobutanol titer of 911 mg l^−1^ was obtained after 46 days with a maximal isobutanol production rate of 43.6 mg l^−1^ day^−1^ observed between days 4 and 6. More frequently, maybe even continuously, removal of the produced isobutanol will further increase both the total titer as well as the rate of production. We also observed that the expression level of Kivd^S286T^ should be kept as high as possible in *Synechocystis* to enhance isobutanol production. Finally, the present study suggested that IlvC and IlvD (heterogenous or endogenous) may be expressed together for further enhancement of isobutanol production. In conclusion, this study provides more detailed metabolic engineering possibilities for direct photosynthetic isobutanol production in *Synechocystis*.

## Additional file

**Additional file 1: Figure S1.** Growth of the *Synechocystis* PCC 6803 empty vector control strain under different light intensities and with different pH adjustments. Results represent the mean of three biological replicates, error bars represent standard deviation. **Figure S2.** Tolerance test for isobutanol and isobutyraldehyde. Three isobutanol concentrations and two isobutyraldehyde concentrations were tested in plug-sealed tissue flasks. The chemicals were added into pEEK2-ST cultures with OD_750_ = 0.5. The change on OD_750_ in each culture after 24 h cultivation was analyzed here. Results represent the mean of three biological replicates, error bars represent standard deviation. **Figure S3.** Schematic overview of the constructs for identifying potential bottlenecks. All the constructs are generated on the self-replicating vector pEEK2. A: Schematic overviews of the constructs with single operon expressing Kivd^S286T^ and one of the respective genes to be examined. B: The construct generated to reduce the expression level of AlsS. C: Schematic overview of the constructs with two convergent orientated operons with a double terminator BBa_B0015 in between.
