# Supplementary material for: Enhancement of photosynthetic isobutanol production in engineered cells of Synechocystis PCC 6803
Source: Biotechnol Biofuels. 2018 Sep 27;11:267. doi: 10.1186/s13068-018-1268-8 (PMC6158846; doi:10.1186/s13068-018-1268-8)
Supplement: Supplementary file 1 — Additional file 1: Figure S1. Growth of the Synechocystis PCC 6803 empty vector control strain under different light intensities and with different pH adjustments. Results represent the mean of three biological replicates, error bars represent standard deviation. Figure S2. Tolerance test for isobutanol and isobutyraldehyde. Three isobutanol concentrations and two isobutyraldehyde concentrations were tested in plug-sealed tissue flasks. The chemicals were added into pEEK2-ST cultures with OD750 = 0.5. The change on OD750 in each culture after 24 h cultivation was analyzed here. Results represent the mean of three biological replicates, error bars represent standard deviation. Figure S3. Schematic overview of the constructs for identifying potential bottlenecks. All the constructs are generated on the self-replicating vector pEEK2. A: Schematic overviews of the constructs with single operon expressing KivdS286T and one of the respective genes to be examined. B: The construct generated to reduce the expression level of AlsS. C: Schematic overview of the constructs with two convergent orientated operons with a double terminator BBa_B0015 in between. [file 13068_2018_1268_MOESM1_ESM.pdf]

Additional file 1

Enhancement of photosynthetic isobutanol production in engineered cells of *Synechocystis* PCC 6803

Rui Miao, Hao Xie, Peter Lindblad*

Microbial chemistry, Department of Chemistry-Ångström Laboratory, Uppsala University, Box 523, SE-751 20 Uppsala, Sweden

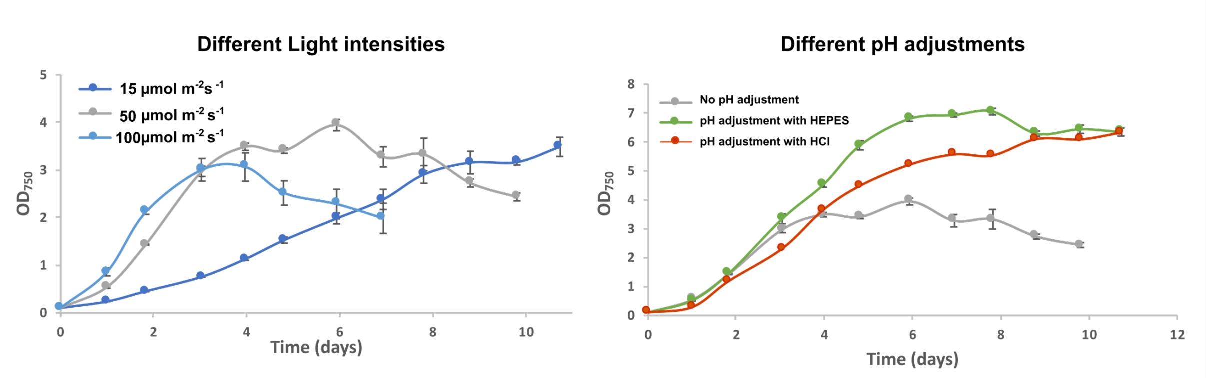

Additional file 1: Figure S1. Growth of the *Synechocystis* PCC 6803 empty vector control strain under different light intensities and with different pH adjustments. Results represent the mean of three biological replicates, error bars represent standard deviation.

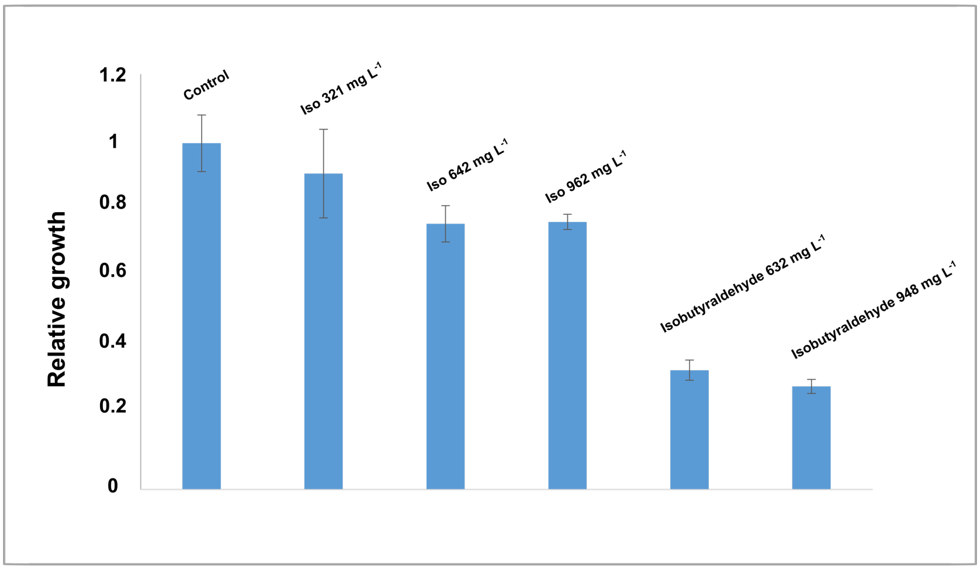

Additional file 1: Figure S2. Tolerance test for isobutanol and isobutyraldehyde. Three isobutanol concentrations and two isobutyraldehyde concentrations were tested in plug-sealed tissue flasks. The chemicals were added into pEEK2-ST cultures with OD_{750} = 0.5. The change on OD_{750} in each culture after 24 hours cultivation was analyzed here. Results represent the mean of three biological replicates, error bars represent standard deviation.

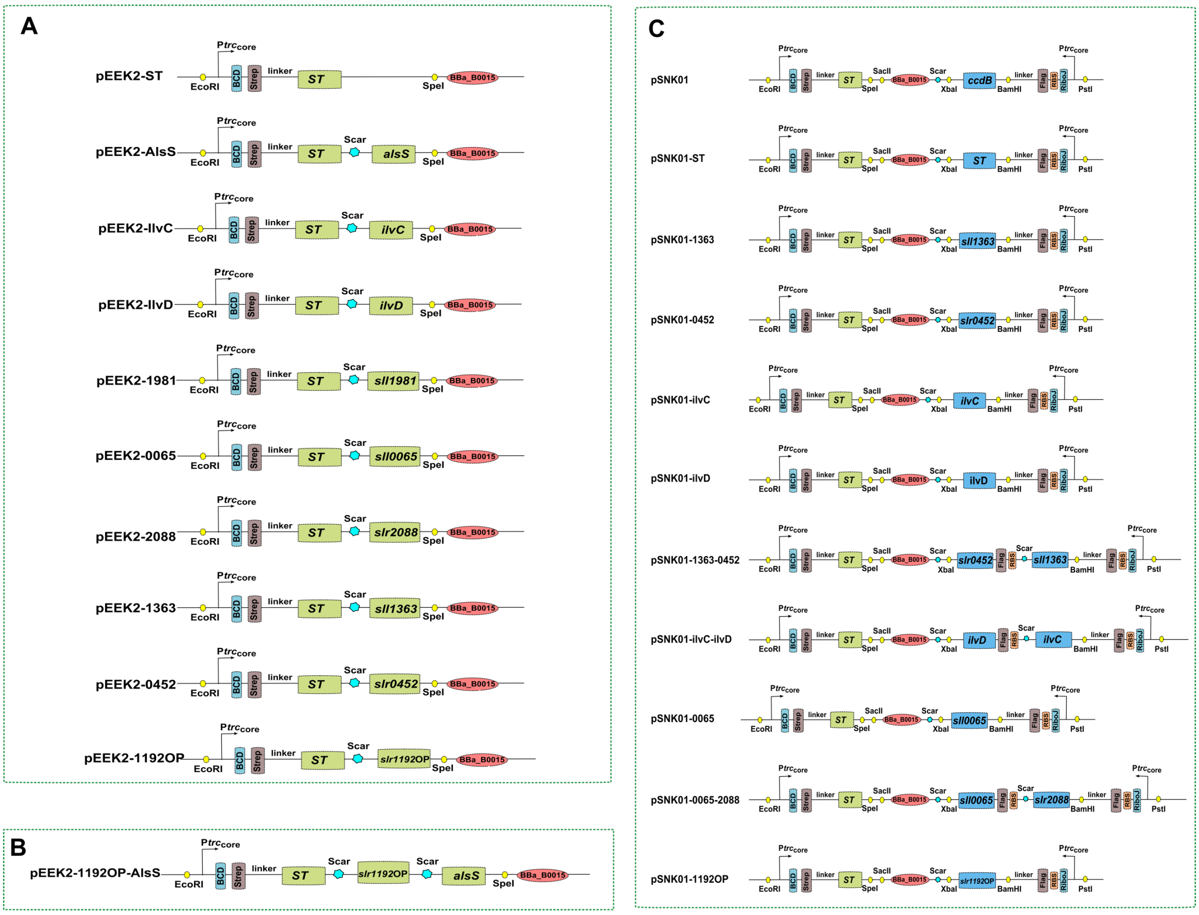

Additional file 1: Figure S3. Schematic overview of the constructs for identifying potential bottlenecks. All the constructs are generated on the self-replicating vector pEEK2. **A**: Schematic overviews of the constructs with single operon expressing Kivd^{S286T} and one of the respective genes to be examined. **B**: The construct generated to reduce the expression level of AlsS. **C**: Schematic overview of the constructs with two convergent orientated operons with a double terminator BBa_B0015 in between.
